# On the Use of Cold Water in Gout

**Published:** 1803-04-01

**Authors:** 


### *To the Editors of the Medical and Phyſical Journal,*

GENTLEMEN,

PERMIT me to say that Dr. Kinglake's use of cold water in the gout is not so new and peculiar to himself as he appears to imagine. The illustrious Harvey, discoverer of the circulation, used to plunge his feet into cold water to mitigate the severity of painful paroxysms of that disease. It has even been said, that he shortened his life by that practice.

As Dr. K. calls loudly upon his medical brethren to communicate any observations which may enable him to make up his mind as soon as possible, the following case, on the authenticity of which he may depend, is much at his service.

The late celebrated Dr. Gregory of Edinburgh, father of the present Professor of Medicine in that University, was very liable to gout. A friend of the present writer called on him one evening, and found him bathing his feet in cold water. He observed to the Doctor, that he was doing what he would hardly recommend to his patients. No, said the Doctor, but this application mitigates pain, which I am unwilling to bear, and I have hitherto experienced no bad effects from it. The next morning the Doctor, to the regret of every admirer of science, and of professional liberality, was found lifeless in his bed.

But why trouble ourselves any farther about the Gout?

One

One gentleman can completely pump out the gout from the system in a day or two! and another has discovered a remedy, a few spoons full of which will enable a cripple to rise from his bed, put on a pair of tight shoes, eat a good dinner, and afterwards dance a hornpipe. Surely, one remedy for a disease is sufficient; my only fear is, that unless we can find out some new diseases, for which as yet there are no remedies, the Faculty must starve!

A CONSTANT READER.

